# Molecular mechanisms of excitotoxicity and their relevance to the pathogenesis of neurodegenerative diseases—an update

**DOI:** 10.1038/s41401-025-01576-w

**Published:** 2025-05-19

**Authors:** Wei-long Wu, Xiao-xi Gong, Zheng-hong Qin, Yan Wang

**Affiliations:** Department of Pharmacology, College of Pharmaceutical Sciences, Suzhou Key Laboratory of Aging and Nervous Diseases, and Jiangsu Key Laboratory of Drug Discovery and Translational Research for Brain Diseases, Soochow University, Suzhou, 215123 China

**Keywords:** neurodegenerative diseases, excitotoxicity, Ca^2+^ homeostasis, oxidative stress, ferroptosis

## Abstract

Glutamate excitotoxicity is intricately linked to the pathogenesis of neurodegenerative diseases, exerting a profound influence on cognitive functions such as learning and memory in mammals. Glutamate, while crucial for these processes, can lead to neuronal damage and death when present in excessive amounts. Our previous review delved into the cascade of excitotoxic injury events and the underlying mechanisms of excitotoxicity. Building on that foundation, this update summarizes the latest research on the role of excitotoxicity in neurodegenerative diseases such as Alzheimer’s disease, Parkinson’s disease, Huntington’s disease, and amyotrophic lateral sclerosis, as well as new cutting-edge techniques applied in the study of excitotoxicity. We also explore the mechanisms of action of various excitotoxicity inhibitors and their clinical development status. This comprehensive analysis aims to enhance our understanding of the nexus between excitotoxicity and neurodegenerative diseases, offering valuable insights for therapeutic strategies in these conditions.

## Introduction

Glutamate is an important excitatory neurotransmitter in the mammalian brain [1, 2]. However, excessive glutamate release can cause over-activation of neuronal glutamate receptors, leading to neuronal oxidative stress [3, 4], mitochondrial damage [5], and disruption of Ca^2+^ homeostasis [6, 7]. These processes exacerbate the effects of glutamate excitotoxicity in neurodegenerative diseases.

As early as 2009, we briefly discussed the relationship between excitotoxicity and neurodegenerative diseases [8]. In the past dozen years, the research on excitotoxicity has continued, and some novel research methods or exploration perspectives have emerged. For example, excitatory/inhibitory balance has been recognized as central to communication between neurons. The inhibitory neurotransmitter γ-aminobutyric acid (GABA), small extracellular vesicles treatment of APP/PS1 mice reduces amyloid β (Aβ) deposition in the brain and improves spatial learning impairment in mice [9]. A recent study has found that 25 Hz repetitive transcranial magnetic stimulation alleviates glutamate excitotoxicity and upregulates the expression of glutamate transporter-1 (GLT-1) by activating phosphatidylinositol-3-kinase (PI3K)/protein kinase B (AKT) in 3xTg AD mice [10]. In addition, intermittent food deprivation enhances hippocampal synaptic plasticity by promoting the expression of sirtuin 3 (SIRT3), thereby maintaining neuronal metabolic balance and reducing excitatory stress and improving the learning and memory ability of App^NL-G-F^ mice [11]. Interestingly, a new concept, “degenerative excitotoxicity,” has been proposed, which may be one of the pathogenic factors of sporadic Alzheimer’s disease (AD). It is understood that there will be no obvious damage in the brain area in the early stage of Ca^2+^ imbalance, but a series of serious and persistent Ca^2+^-dependent cytotoxicity will occur later [12]. Initially, excitotoxicity can be effectively mitigated, yet with the passage of time, its deleterious influence progressively intensifies, culminating in the intractability of neurodegenerative diseases.

Strict regulation of glutamate levels in the synaptic cleft is very important to maintain the normal physiological function of neurons. In certain pathological or abnormal physiological states, this balance may be disturbed. Overexcitation of the neuron leads to depolarization of the presynaptic membrane, prompting the opening of voltage-gated Ca^2+^ channels and allowing a massive influx of Ca^2+^ into the presynaptic neuron [13–15]. Abnormal expression or altered function of glutamate receptors and changes in the subunit composition of the receptors lead to increased sensitivity of neurons to glutamate, prompting the release of more glutamate [16–18]. When energy supply is low, intracellular glutamate leaks outside the cell due to failure of energy-dependent transport mechanisms [19]. When glial cells function abnormally, their ability to uptake and metabolize glutamate is reduced, and they are unable to efficiently clear glutamate from the synaptic cleft [20]. Mutations in related genes can directly affect synaptic structure and function [21]. Disease-associated proteins also interfere with intracellular signaling pathways, such as Aβ [22]. When excessive glutamate is released from the presynaptic, it stimulates the postsynaptic ionotropic glutamate receptors (iGluRs) and metabotropic glutamate receptors (mGluRs). It ultimately affects the balance of glutamate between synapses and neurons, leading to neuronal excitotoxic death. In conclusion, excessive glutamate release is a multifactorial combination of factors that affect the nervous system.

Here, we provide a succinct overview of the excitotoxicity phenomenon and the repercussions of the aberrant hyperactivation of glutamate receptors. Furthermore, we elucidate the contemporary molecular underpinnings of glutamate-driven excitotoxicity. Subsequently, we delve into the therapeutic interventions for glutamate-induced excitotoxicity in the context of neurodegenerative afflictions that have emerged during the preceding decade. Such an examination is instrumental for enhancing our comprehension of the nexus between excitotoxicity and the etiology of neurodegenerative diseases.

## Recent progress in the regulation of glutamate receptors

Neuronal excitotoxicity involves prolonged glutamate exposure, triggering excessive ion influx, leading to neuronal death [23]. As the brain’s primary excitatory neurotransmitter, hippocampal glutamate regulates synaptic plasticity essential for learning and memory [24]. There are two classes of glutamate receptors, iGluRs and mGluRs. Both receptors are active in presynaptic, astrocytic, and postsynaptic receptors. mGluRs have eight different subtypes, some of which can be coupled to adenylate cyclase and phospholipase C. iGluRs are classified into three types: N-methyl-D-aspartate (NMDA) receptors, kainate receptors (KARs), and α-amino-3-hydroxy-5-methyl-4-isoxazole propionate (AMPA) receptors.

### Metabotropic glutamate receptors

Glutamate activates mGluRs after synaptic release, which are widely expressed in the peripheral and central nervous systems (CNS) and play multiple neuroregulatory roles [25–28]. Based on the expression, amino acid sequence, and functional mechanism of the eight mGluRs subtypes, they are divided into three groups: Group I: mGluR1 and mGluR5, Group II: mGluR2 and mGluR3, and Group III: mGluR4 and mGluR6-8 [29, 30].

The mGluR1 accumulates in dendrites and is mainly localized in the plasma membrane to regulate neuronal functions. The mGluR1α is expressed in the hippocampus and entorhinal cortex of patients with AD. The 3,3′-diaminobenzidine immunoreactivity analysis of mGluR1α showed strong diffuse staining in CA1 and CA3 regions, thereby making mGluR1α a therapeutic target for AD [31]. Compared with mGluR1, recent studies have focused more on mGluR5. For example, the 14,15-epoxyeicosatrienoic acid (14,15-EET) is a lipid mediator, the synthesis of which can activate downstream mGluR signaling in astrocytes. Inhibition of soluble epoxide hydrolase induces neuroprotective effects by blocking the degradation of 14,15-EET. The soluble epoxide hydrolase inhibitors (sEHi) 12-(3-adamantan-1-yl-ureido)-dodecanoic acid and 14,15-EET preserve the integrity of astrocytes and mitigate excitotoxicity, and their effects are dependent on mGluR5 [32]. Homer 1a, a postsynaptic scaffold protein, protects against traumatic brain injury by regulating mGluR1 and mGluR5. Overexpression of Homer 1a attenuates the protective effect of mGluR5 agonists on traumatic injury, whereas its knockdown improves neuronal injury after activating mGluR5 [33]. Conventional signaling of mGluR1/5 is delivered via G protein coupling, but studies have suggested that they may act via non-classical pathways. For example, after knockout of β-arrestin 2, mGluR1/5-mediated plasticity of CA3 and CA1 pyramidal neurons was impaired, suggesting that there is a non-classical signaling pathway dependent on β-arrestin 2 in mGluR1 [34]. According to positron emission tomography, mGluR5 is mainly located in the postsynaptic striatum, specifically in D2 neurons; therefore, mGluR5 levels may reflect whether the striatum is degenerated [35, 36]. mGluR5 acts as an important cofactor, its chronic activation can enhance NMDA receptors (NMDARs) activity and further amplify neuronal excitotoxicity [37].

However, unlike mGluR5 in Group I, activation of mGluR7 in Group III reduced NMDA-mediated currents and NR1 surface expression in rodent basal forebrain cholinergic neurons through the regulatory mechanism of cofilin (CFL). But its protective effect can be selectively disrupted by Aβ [38]. The mGluR7 allosteric agonist AMN082 attenuates oxygen glucose deprivation (OGD)-induced release of lactate dehydrogenase (LDH) in a concentration- and time-dependent manner and protects cortical and hippocampal neurons by restraining caspase-3 [39]. In addition, in a chronic dopamine-deficient Parkinson’s disease (PD) model, activation of astrocytes external globus pallidus externus can restore the activity of mGluR3 and significantly inhibit the release of striatal pallidal GABA. This suggests that targeting astrocyte glutamate metabolism or metabolic modulators of mGluR3 may be a new strategy to alleviate PD motor symptoms [40]. Overall, these results demonstrate that the normal expression of mGluRs can promote the development of neurons, synaptic plasticity, and learning and memory ability.

### Ionotropic glutamate receptors

The NMDARs are a major subtype of iGluRs that are involved in synaptic plasticity, development of neuronal dendritic structure, and learning and memory processes. NMDAR subunit 2 (NR2) is an important subunit of NMDAR, which has four different subtypes: NR2A, NR2B, NR2C, and NR2D. A recent study found that the absence of NR2A in the brain of adult mice triggers antidepressant-like behavior, and ketamine can increase the intrinsic excitability of hippocampal neurons by inhibiting NR2A [41]. NR2C hierarchically modulates synaptic strength in CA1 pyramidal neurons, where its inhibition narrows presynaptic strength distribution in radial inputs, impairing long-term synaptic plasticity and computational functions [42]. In addition, compound YY-23 targets prefrontal cortical GABAergic interneuron NR2D-NMDARs, suppressing inhibitory GABAergic transmission while boosting excitatory signals to exert rapid antidepressant effects [43]. Glutamate-mediated excitotoxicity deforms dorsal horn neurons; however, the conditional deletion of *Grin1*, a subunit of NMDARs, prevents the loss of these neurons [44]. Disulfide high mobility group box-1 dose-dependently increases the phosphorylation of Tyr^1472^ of NR2B and enhances the expression of toll-like receptor 4 (TLR4), which is associated with permeability of Ca^2+^ channels in hippocampal neurons [45]. Glutamate exposure in rat hippocampal neurons triggers a sustained elevation of extracellular adenosine triphosphate (ATP) levels, subsequent activation of P2Y1 receptors (P2Y1R), and ultimately results in hippocampal neuron death [46]. The N-arachidonoylphenolamine dose-dependently inhibits NMDA-induced excitotoxicity in organotypic hippocampal slice cultures and the expression of interleukin-6 (IL-6), tumor necrosis factor α (TNF-α), and microsomal prostaglandin E synthase-1 and attenuates neuronal damage [47]. Dual leucine zipper kinase interacts with postsynaptic density protein-95 (PSD-95), and the uncoupling of NMDAR and PSD-95 can rescue NMDA-mediated neurotoxicity damage [48, 49].

The AMPA receptors (AMPARs) consist of four distinct subunits: GluA1, GluA2, GluA3, and GluA4; they are highly expressed in the hippocampus of mammals and are extremely vital for maintaining long-term potentiation, thereby transmitting excitatory signals and improving memory. The NMDAR is more widely distributed in the synaptic membrane and cytoplasm of pyramidal cells in the hippocampal CA1 region than AMPARs. Nevertheless, changes in the composition of glutamate receptors may alter the properties of glutamate receptor channels because AMPARs lack GluR2 and are more sensitive to excitotoxicity [50]. The secretion factor of mesenchymal stem cell-conditioned medium (MSC-CM) decreases the cell surface expression of the GluA1 subunit of AMPAR in cortical neurons. Immunofluorescence results showed that GluA1 is located in the nucleus rather than evenly dispersed in the cytoplasm. Therefore, the secretion factor of MSC-CM exerts a neuroprotective effect. However, acidification did not increase or decrease the expression of GluA1 in neurons [51]. There is a piece of evidence that continuous infusion of AMPA in the lumbar spinal cord of adult rats resulted in excessive activation of Ca^2+^-permeable AMPARs (CP-AMPARs), leading to progressive hindlimb paralysis and bilateral motor neuron (MN) degeneration [52]. Another study found that OGD activates acid-sensing ion channel 1a (ASIC1a)-dependent CP-AMPAR expression, which contributes to cellular acidosis and excitotoxicity in the hippocampal CA1 region. However, the combined inhibition of ASIC1a and CP-AMPARs is insufficient to restore the normal activity of neurons and ASIC1a and CP-AMPARs may thus be drug targets for neuroprotection [53].

KARs are tetramers composed of five distinct subunits (GluK1–5). KA is not only a complete agonist of KARs but also a partial agonist of AMPARs [54, 55]. Accumulating evidence indicates that KA activates KAR-mediated epilepsy and excitotoxicity [56–58]. Systemic KA administration can induce extensive neuronal loss in CA1, whereas intracerebral KA injection can cause neuronal loss, mainly in the hippocampal CA3 region [59, 60]. A previous study has also demonstrated that systemic administration of KA induces contraction and even death of piriform cortical neurons. The KA causes progressive motor epilepsy symptoms in rodents, such as walking imbalance, forelimb twitching, and hindlimb standing [61]. It also causes various molecular and cellular changes, such as changes in axons and dendrites, expression of caspase-3 and B cell lymphoma-2 (Bcl-2) [62], and, in severe cases, neurodegeneration. Parkin interacts with the GluK2 subunit of KARs. Because Parkin and GluK2 co-immunoprecipitate and Parkin ubiquitinates GluK2, the loss of Parkin function leads to GluK2 accumulation in the cell membrane [63]. Interestingly, KA influences cell cycle reentry (CCR) through the Notch mechanism to induce errors and facilitate neuronal death. While KA activates Notch signaling, activity of CyclinD1 is increased through AKT/glycogen synthase kinase-3β (GSK-3β) signaling pathway, which leads to the transformation and abnormalities in G_1_-S in CCR [64]. Injection of TLR2 agonist Pam3CSK4 or TLR4 agonist lipopolysaccharide (LPS) before the striatal injection of KA significantly activated microglia and protected cortical neurons. Mice lacking myeloid differentiation factor-88 were more susceptible to KA-mediated excitotoxicity than wild-type mice [65]. Over the past decade, P2Y1R could be considered a catalyst for reducing the threshold of glutamate excitotoxicity. P2Y1R inhibition significantly alleviated KA toxicity in hippocampal neurons, thus indirectly exerting a neuroprotective effect [66]. In short, compared with mGluRs, iGluRs seem to be more critical in excitotoxicity.

## Process and mechanism of multimodal cell death in excitotoxicity

Glutamate-mediated excitotoxicity triggers neuronal damage through multi-cascade pathological mechanisms. The initiating event involves Ca^2+^ homeostasis disruption leading to intracellular Ca^2+^ overload. Sustained over-activation of glutamate receptors subsequently induces synergistic injury mechanisms, including oxidative stress, mitochondrial metabolic dysfunction, endoplasmic reticulum (ER) stress, and nicotinamide adenine dinucleotide (NAD^+^) homeostasis disruption. These pathological interwoven processes activate multimodal cell death pathways, such as apoptosis, necrosis, autophagy, and ferroptosis, posing a major challenge to neuroprotective treatment strategies.

### Cascade process of excitotoxic neuronal death

**Fig. 1:**
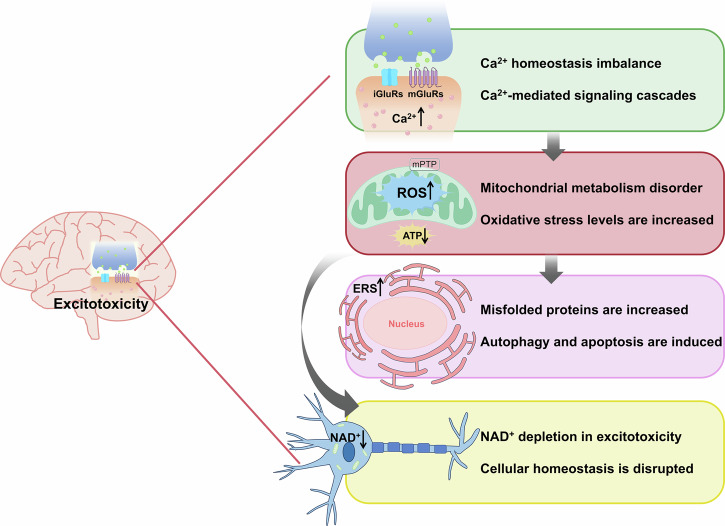
Molecular mechanisms mediated by excitotoxicity. The excitotoxicity-induced pathological process in neurons represents a complex cascade with extensive crosstalk among cellular systems. Firstly, glutamate over-activation of ionotropic glutamate receptors (iGluRs) and metabotropic glutamate receptors (mGluRs) triggers massive Ca²⁺ influx, disrupting dynamics of glutamate receptors and intracellular Ca²⁺ homeostasis. Secondly, mitochondrial Ca²⁺ overload activates mitochondrial permeability transition pore (mPTP) opening, changes Δ*Ψ*_m_, and triggers reactive oxygen species (ROS) production. Drp1-mediated excessive division and PINK1/Parkin pathway-mediated mitophagy lead to mitochondrial dynamic imbalance and reduced adenosine triphosphate (ATP) production. ATP deficiency affects both endoplasmic reticulum stress (ERS) and intracellular nicotinamide adenine dinucleotide (NAD^+^) metabolism. On the one hand, because the ER needs ATP for protein folding, ATP deficiency activates UPR and protein misfolding, resulting in ERS. On the other hand, ATP reduction leads to the activation of PARP-1 to participate in repair. However, activation of PARP-1 will consume a large amount of NAD^+^, and eventually, the intracellular NAD^+^ metabolism is unbalanced.

Excessive Ca²⁺ influx disrupts Ca²⁺ homeostasis, overwhelming mitochondrial buffering via the uniporter. This causes mitochondrial membrane depolarization, mitochondrial permeability transition pore (mPTP) opening, and dysfunction. Concurrently, dynamin-related protein 1 (Drp1)-mediated excessive fission and impaired PINK1/Parkin-dependent mitophagy exacerbate mitochondrial damage [67, 68], while elevated oxidative stress markers amplify injury. ATP depletion activates ER stress (ERS) pathways [69, 70], leading to misfolded protein aggregation and redox imbalance if unresolved. NAD⁺ depletion from poly (ADP-ribose) polymerase-1 (PARP-1) hyperactivation further impairs SIRT function, mitochondrial biosynthesis, and antioxidant defenses [71, 72]. These interconnected axes-Ca²⁺ dysregulation, mitochondrial collapse, ERS, and NAD⁺ crisis form a self-amplifying loop that highlights multi-target therapeutic opportunities for neurodegenerative diseases (Fig. 1).

#### Abnormal dynamics of glutamate receptors

Neurons in the normal environment can maintain intracellular and extracellular glutamate levels by relying on the interconnection between proteins. However, impaired glutamate uptake breaks this homeostasis, resulting in neuronal excitotoxicity, changes in synaptic plasticity, dysfunction of astrocytes and microglia, and neurodegeneration [73, 74]. In pursuit of alleviating cell damage caused by glutamate-induced excitotoxicity, current drug research mainly focuses on NMDAR antagonists. S-sulfocysteine can function as an NMDAR agonist to aggravate sulfite neurotoxicity in the brain, promote depolarization of presynaptic membrane sites, and induce a large Ca^2+^ influx [75]. Interestingly, a recent study has shown that simultaneously blocking NMDAR and PARP-1 activities can effectively mitigate 3-nitropropionic acid (3-NP)-induced HD in a mouse model [76].

When conventional wisdom is bypassed, many unexpected positive outcomes often emerge. NR2A and NR2B are two subunits of NMDAR, and there is an 18 amino acid fragment called I4 on the inner membrane of their cells. I4 can interact with a 57-amino acid intracellular domain of transient receptor potential melastatin 4 (TRPM4). After the NMDAR and TRPM4 form a complex, the neurons are more vulnerable to excitotoxicity. It has become a hot spot to break the interaction of the NMDAR/TRPM4 complex and study the interface inhibitors of NMDAR/TRPM4 interaction [77]. FP802 is a TwinF interface inhibitor that alleviates glutamate neurotoxicity mediated by extrasynaptic NMDARs (eNMDARs) by disrupting the NMDAR/TRPM4 complex, but does not affect the normal physiological function of synaptic NMDARs [78]. There is also an interaction between TRPM2 and NMDAR. TAT-M2PBM, a peptide that disrupts TRPM2-protein kinase C γ (PKCγ), a cell-permeable peptide that disrupts TRPM2-PKCγ coupling, significantly inhibits eNMDARs-mediated excitotoxicity and produces potent protection against ischemic stroke in vitro and in vivo [79]. To recapitulate, precise regulation of glutamate receptors by protein structure or administration may be a potential strategy to alleviate excitotoxicity.

#### Ca^2+^ homeostasis imbalance

Upon the release of the excitatory neurotransmitter glutamate from the presynaptic terminal, it initiates the activation of postsynaptic receptors, thereby inducing alterations in the electrical potential across the postsynaptic membrane. Concurrently, there is an influx of Ca^2+^ into the neuronal compartment through these receptors, which subsequently engage in the modulation of a plethora of metabolic and anabolic processes [80]. Recent developments in research techniques, such as single-cell RNA-sequencing, have accurately dissected gene expression changes following NMDAR-induced injuries in the mouse retina, clarifying the role of microglia in regulating inflammatory cytokines [60]. Notably, microRNAs play critical roles in modulating synaptic receptors. Overexpression of miR-146a and miR-200b downregulates NR2A and NR2B proteins, contributing to cognitive deficits [71], while miR-137 controls excitatory synapse maturation and plasticity by regulating GluA1 expression, with its levels modulated by mGluR5 activation [63]. Advanced imaging techniques like event-triggered STED further elucidate these dynamics; this adaptive microscopy method combines rapid wide-field imaging with targeted STED to detect cellular events and initiates 3D imaging within 40 ms, enabling precise observation of synaptic protein reorganization during neuronal Ca^2+^ activity [81].

However, the sustained and excessive release of glutamate leads to a large influx of Ca^2+^, which disrupts Ca^2+^ homeostasis and damages the mitochondrial function of neurons. In more serious cases, it can also cause neuronal death [82, 83]. Therefore, it is particularly vital to maintain the balance of Ca^2+^ in neurons. In the brain of GluN3A knockout mice, some Ca^2+^-regulated signaling molecules, such as Ca^2+^-calmodulin (CaM)-dependent protein kinase II (CaMKII), PKCα, and calpain, are increased. The consistent increase of Ca^2+^ in neurons aggravates Aβ deposition and Tau protein phosphorylation [9]. High intracellular reactive oxygen species (ROS) and Ca^2+^ levels activate AMP-activated protein kinase (AMPK), which is necessary for the KA-induced apoptosis of hippocampal neurons [64]. In zebrafish larvae, exposure to exogenous AMPA induces Ca^2+^ inflow, whereas blocking CP-AMPARs effectively protects the terminals from Ca^2+^-mediated excitotoxic damage [84]. Astaxanthin can reduce the expression of NMDARs and AMPARs, regulate Ca^2+^ dynamics, and inhibit abnormal depolarization of mitochondrial membrane potential (Δ*Ψ*_m_) and ROS production [85, 86]. Recent studies have demonstrated that the anthocyanins obtained from Korean black beans may attenuate KA-induced Ca^2+^ imbalance, AMPK and caspase-3 activation, cytochrome-*c* release, and decreased Bcl-2 levels in HT22 cells [87]. In aggregate, the onset of excitotoxicity is precipitated by a perturbation in Ca^2+^ homeostasis. Subsequently, an ensemble of Ca^2+^-mediated cascade reactions exacerbates the excitotoxic cascade. Ca^2+^ overload can trigger the activation of multiple signaling pathways, such as the calcineurin/nuclear factor of activated T-cells pathway [88] and the cyclic adenosine monophosphate/protein kinase A pathway [89], in addition to exerting significant effects on mitochondrial function.

#### Oxidative stress and mitochondrial dysfunction

Glutamate is the dominant cause of oxidative stress in the CNS via two mechanisms. The first is the glutamate receptor mechanism, which induces ROS production by activating calpain 1, phospholipase A2, and nitric oxide synthase (NOS) after glutamate binds to NMDARs [90]. The second mechanism is through the interaction between glutamate and the cystine transporter, which reduces intracellular glutathione (GSH) and increases ROS accumulation. Superoxide anion radical (O_2_•−), hydrogen peroxide (H_2_O_2_), hydroxyl radical (HO•), and singlet oxygen (^1^O_2_) are the sources of ROS in neurons. Oxidative and nitrosative stress are the predominant contributors to neuronal death in neurodegenerative diseases.

ROS are mediators of oxidative stress, and some evidence suggests that they are significantly involved in oxidative stress that activates the mechanisms of excitotoxicity and neurodegeneration in different brain regions [91]. ROS accumulation destroys the mitochondrial membrane and induces nerve deformation and cell apoptosis. Some apoptotic genes, like *caspase-3*, *caspase-6*, *c-Jun nh2-terminal kinase* (*JNK*), etc., their activity has also been severely affected. Hypoxia/reoxygenation (H/R) leads to excessive ROS production in primary hippocampal neurons [92]. Whereas agar in red algae can protect cells from H/R-induced DNA damage, reduce ROS production, and stabilize Δ*Ψ*_m_ [93]. Coenzyme Q10 is similar in structure to vitamin E and acts as a mitochondrial energy converter. Its role is to maintain Δ*Ψ*_m_ and support and inhibit ATP synthesis, thereby effectively protecting neurons [94–98]. Nuclear factor erythroid 2-related factor 2 (Nrf2) is instrumental in mitigating neuronal oxidative stress and glutamate-induced excitotoxicity. Counteracting oxidative stress by Nrf2 upregulation is effective for treating neurological disorders [24, 99].

Nitric oxide (NO) is used to initiate defensive protection in mammals and accumulates in the brain [100]. Nevertheless, a marked escalation in NO production by inducible NOS (iNOS) readily incites inflammation within the body [101]. The expression of inflammatory factors may affect glutamate receptors, promote the opening of Ca^2+^ channels, and mitochondrial dysfunction. Activation of NMDARs increases iNOS production, induces the production of a large amount of neurotoxic NO, and, ultimately, gives rise to apoptosis [102, 103]. Preso promotes the signal transduction of NMDAR to NO and Ca^2+^ response by regulating the NR2B/PSD-95/nNOS complex. Conversely, the knockdown of Preso expression alleviated Ca^2+^ overload and NO production [104–107]. The direct interaction between nNOS and sex determining region Y-box 2 in the hippocampus of rats after middle cerebral artery occlusion (MCAO) is significantly improved by co-immunoprecipitation and the promotion of sonic hedgehog transcription as a new feedback compensation mechanism for protecting neurons [108]. Some researchers have also been attracted by traditional Chinese medicine in alleviating NO-mediated neuroinflammatory response. Icariin treatment significantly inhibited the activity of apoptotic factors caspase-3 and Bcl-2-associated X protein (Bax)/Bcl-2, and reduced intracellular ROS and NO levels [67]. *Aquilariae Lignum* fraction pretreatment of LPS-stimulated BV2 microglia cells significantly reduced NO, cyclooxygenase-2 (COX-2), IL-1β, and prostaglandin E2 levels and attenuated nuclear factor-kappaB (NF-κB) and p65 activities [109].

#### Endoplasmic reticulum stress and unfolded protein response

As an intracellular organelle, the ER is important for protein folding, transportation, and quality control. However, when cells are exposed to harsh environments, such as oxidative stress and impaired Ca^2+^ homeostasis, resulting in an increase in unfolded proteins, triggers ERS. In response to ERS, cells trigger an unfolded protein response (UPR). UPR has three important sensors, namely protein kinase R-like ER kinase (PERK), inositol requiring enzyme 1 (IRE1), and activating transcription factor 6 (ATF6), which activate ERS markers C/EBP homologous protein (CHOP) and caspase-12 after separation [110, 111]. ERS causes a complete imbalance of intracellular Ca^2+^ homeostasis, and the UPR of the cell itself loses its function, eventually leading to apoptosis and autophagy.

Studies have found that KA induces ERS through G protein inwardly rectifying K^+^ receptor, activates ATF6, glucose-regulated protein 78 and IRE1, and stimulates the expression of pyrin domain-containing protein 3 (NLRP3) and NF-κB [111]. ATF6 is closely related to calreticulin expression. The study found that ATF6 deficiency reduced Ca^2+^ storage in the ER, causing a temporary decrease in the mRNA levels of *calnexin*, *GRP94*, and *GRP7*. After administration of KA, ERS is significantly enhanced in ATF6^−/−^ neurons [112]. When protein misfolding occurs, there is another way of cell protection called the integrated stress response (ISR). Nonetheless, NMDA-induced ISR is an ATF4-independent dephosphorylation of eukaryotic translation initiation factor 2α (eIF2α) [113]. The depletion of ER Ca^2+^ promotes the formation of mitochondrial-associated ER membrane, leading to mitochondrial Ca^2+^ overload and dysfunction. Sarco/ER Ca^2+^-ATPase 2b (SERCA2b) can transport Ca^2+^ from the cytoplasm to the ER. SERCA2b mutation accelerates the depletion of ER Ca^2+^, promotes ERS, and aggravates the stimulation of glutamate to HT22 cells’ excitotoxicity [114]. The expression of ERS genes *caspase-12*, *ATF4*, and *CHOP* in MCAO rats increases, and the levels of apoptosis increase. After combined treatment with ginsenoside Rg1 and mannitol, p-PERK, p-eIF2α, and ATF4 are downregulated, which effectively alleviates glutamate-induced neuronal ERS [115]. In summary, these findings demonstrate that excitotoxicity induces neuronal ERS and UPR. Addressing excitotoxicity at the subcellular level mirrors a regulatory approach that spans from the microscopic to the macroscopic domain, thereby conferring a degree of therapeutic relevance.

#### NAD^+^ metabolic disturbance

NAD^+^ has neuroprotective effects in acute axonal injury, chronic nerve stress, and different neurodegenerative diseases associated with excitotoxicity [116]. The imbalance of energy metabolism caused by excitotoxicity is also one of the pivotal causes of cell death. Moreover, NAD^+^ depletion is very common in excitotoxicity models [117]. Therefore, to maintain cellular energy homeostasis, supplementation of NAD^+^ and nicotinamide adenine dinucleotide-reduced (NADH) coenzymes is necessary. Supplementation of NAD^+^ in primary cortical neurons can effectively alleviate glutamate-induced Δ*Ψ*_m_ depolarization, mitochondrial damage, and NADH redistribution. This process may play an indispensable role in mitochondrial protection by activating SIRT1. Moreover, supplementation of NAD^+^ can effectively reduce neuronal apoptosis and translocation of apoptosis-inducing factors after glutamate stimulation [118, 119]. Nicotinamide mononucleotide adenosine transferase (NMNAT) is one of the loops in the synthesis of NAD^+^. NMNAT3 is the main NMNAT subtype in mitochondria. Overexpression of NMNAT3 reduced the activity of calpain and caspase-3, decreased the degradation of calpastatin, and alleviated the damage of the cortex and hippocampus in mice [120].

Excitotoxicity of glutamate has a direct effect on the inflammatory injury of neurons [121]. Activation of the NMDAR causes the nicotinamide adenine dinucleotide phosphate (NADPH) oxidase to produce superoxide, which conducts signal transduction between neurons. The combination of NADPH and Mito-apocynin, a mitochondria-specific NADPH oxidase (NOX) inhibitor, effectively reversed KA-induced neuronal damage and the down-regulation of TP53-induced glycolysis and apoptosis regulator and NOX4 [122]. In addition, NAD^+^ is a substrate for many cellular enzymatic reactions, such as PARP-1 and the deacetylase family SIRTs. In the NMDA-induced olfactory bulbs (OB) excitotoxicity model, the expression of SIRT1 and SIRT4 in OB increased, while the expression of SIRT2 did not increase, which may be related to the recovery of olfactory function [123]. NAD^+^ can inhibit the release of presynaptic glutamate, reduce the induced excitatory postsynaptic current, and weaken excitatory neurotransmission, thereby inhibiting bilirubin-induced hyperexcitability of ventral cochlear nucleus neurons [124]. In conclusion, the metabolic pathway of NAD^+^ is essential for the preservation of neuronal energy homeostasis.

### Multimodal cell death in excitotoxicity

Various forms of cell death are observed in neurodegenerative diseases, most commonly cell death caused by glutamate receptor-induced excitotoxicity, including apoptosis, necrosis, autophagy, and ferroptosis. These cell death mechanisms may play opposite roles in different situations, but are nevertheless vital to maintaining organismal metabolic balance and promoting neuroprotection.

#### Apoptosis

Apoptosis can be divided into caspase-dependent and caspase-independent. Specifically, AIF and caspase-3 play critical roles in caspase-independent and caspase-dependent apoptosis, respectively. Bone marrow mesenchymal stem cell-derived exosomes reduce the expression of caspase-3 and caspase-9 [118]. The mGluR5 activation attenuates NMDA-induced excitotoxicity in differentiated PC12 cells by disrupting the NMD-PSD-95 complex, thereby preserving mitochondrial function. Moreover, mGluR5 is activated to inhibit LDH activity, thereby increasing the Bax/Bcl-2 ratio [37]. Apoptotic cell death is usually caused by the activation of caspase-3 [119]. Anthocyanins alleviate the upregulation of Bax, activation of caspase-3, and decrease in Bcl-2 expression in KA-treated HT22 cells [83]. Striatum quinolinic acid not only increases intracellular Ca^2+^ and promotes oxidative stress but also activates different cell death mechanisms by reducing Bcl-2 expression and increasing JNK activity [88]. *O*-Linked *N*-acetylglucosamine (O-GlcNAc) is a dynamic post-translational modification of serine and threonine residues in the nuclear cytoplasm. O-GlcNAc-modified NOS1 adapter (NOS1AP) exerts a protective effect against glutamate-induced neuronal apoptosis because of the decreased interaction between NOS1AP and neuronal nNOS [120].

#### Necrosis

Necrosis is an important pathogenic mechanism of glutamatergic neuronal death in the CNS [121, 122]. Chromatin dysfunction caused by single- or double-strand DNA damage mediated by ROS is associated with cell necrosis. MN disease occurs under excitotoxic conditions induced by acute AMPA. The early stage of this process is characterized by apoptosis, followed by cytoplasmic vacuolization, ER swelling and cell membrane rupture leading to cell necrosis [123, 124]. Blocking the insulin/IGF signaling cascade reduces the susceptibility of cells to excitotoxic necrosis [125]. Cyclic AMP response-binding protein (CREB)/corticotropin-releasing hormone-1, which is produced in wild-type animals, protects neurons from excitotoxic necrosis [14]. In AD, Tau^A152T^-induced glutamatergic neuron loss is mainly caused by necrosis, which disrupts lysosomal catabolic aspartic protease cathepsin D activity [121]. 2-ethyl-6-methyl-3-hydroxypyridinium gammalactone-2,3-dehydro-*L*-gulonat dose-dependently rescues glutamate- or OGD-induced cell necrosis [126]. These results indicate that inhibition of neuronal excitotoxicity can alleviate neuronal necrosis to a certain extent.

#### Autophagy

Autophagy is a major catabolic pathway in organisms and is a crucial cellular process after glutamate-induced excitotoxicity. Increased microtubule-associated protein light chain 3II (LC3-II) levels or LC3-II/LC3-I ratios are commonly observed in cells exposed to NMDA or other glutamate receptor agonists [127]. Excessive autophagy might lead to cell death, which is a new way of cell death in addition to apoptosis and necrosis [128].

As a glutamate receptor agonist, KA can also activate autophagy. A previous study showed that KA variously regulated the expression of autophagy-related proteins depending on dose and treatment frequency. However, NADPH can reverse the changes in KA-induced LC3, sequestosome-1 (p62/SQSTM1), and NOX4 protein expression, reduce autophagosome production, and maintain normal neuronal function [122]. Unc-51-like autophagy activating kinase 1 (ULK1), lysosomal-associated membrane protein 1, and BECN1 (Beclin-1) are also markers of autophagy. Inhibition of autophagy by ULK1 knockdown mitigates neurite rupture in primary cortical neurons affected by glutamate excitotoxicity [128]. Interestingly, glutamate also inhibits hunger-induced autophagy in SH-SY5Y cells. Consequently, the transformation of LC3-I to the autophagosome-associated form LC3-II is weakened, thereby reducing Beclin-1 and autophagy-related gene 5 expression and decreasing autophagy vesicle formation [129]. In the early stage of excitotoxicity, autophagy may play an indispensable role in the “self-rescue” of neurons. As the cellular stress intensifies, the expression of autophagy-related proteins surges once more, reinforcing the cell’s efforts to degrade and recycle cellular components. This autophagic response is a bid to preserve energy homeostasis and stave off the impending demise. In the late stage of disease development, neuronal autophagy may be a process of “self-destruction”, which may be that the intracellular state is beyond the control of neurons.

#### Ferroptosis

Ferroptosis is a newly identified type of programmed cell death characterized by the iron-regulated accumulation of lipid peroxidation. It is initiated by changes in intracellular and extracellular lipid peroxidation levels, the balance in GSH oxidation and reduction, and intracellular iron metabolism abnormalities, which promote CNS damage and neuronal death [130].

Several recent studies have shown that iron chelators, ferrostatin-1 [131], Liproxstatin-1 [132], and other drugs [133] protect against acute stroke and neurodegenerative diseases by inhibiting ferroptosis. This novel pathway adds another layer of complexity to the cell’s struggle against excitotoxicity. Our recent study found that NADPH promotes recruitment of ferroptosis suppressor protein 1 (FSP1) to the plasma membrane through N-myristoyltransferase 2 (NMT2), which plays a vital role in resisting KA-mediated neuronal ferroptosis. Among them, the Arg-291 site of NMT2 is crucial for the neuronal protection of the NADPH-NMT2-FSP1 axis [134]. Cells lacking CFL1 may complement the energy needs of cells by enhancing glycolysis, while normal cells are damaged during iron metabolism. Down-regulation of CFL1 or decreased CFL1 phosphorylation can alleviate erastin-induced HT22 cell death and reduce the glutamate-induced excitotoxicity of primary cortical neurons [135]. Forkhead box class O3 (FoxO3) interacts with solute carrier family 7 member 11 to inhibit its expression and reduce glutamate excitotoxicity. Conversely, the AMPK/FoxO3 signaling pathway regulates mitochondrial activity and alleviates cell damage caused by ferroptosis [136]. Our recent study also found that under the stimulation of KA, the expression of ferritin heavy chain 1 (FTH1) is downregulated and the expression of nuclear receptor coactivator 4 (NCOA4) is up-regulated. Knockdown of NCOA4 in HT22 cells rescues KA-mediated down-regulation of FTH1 expression [137]. At present, more and more studies have shown that the development of excitotoxicity is accompanied by ferroptosis, and their previous regulatory mechanisms have yet to be elucidated.

## Excitotoxicity in neurodegenerative diseases

Neurodegeneration, characterized by progressive neuronal loss, drives incurable disorders like AD, PD, HD, and amyotrophic lateral sclerosis (ALS) [60, 138]. Excitotoxicity is a key neurodegenerative mechanism that disrupts neuronal function and survival in CNS disorders [139]. Over the past decade, research has increasingly focused on unraveling the interplay between excitotoxicity and neurodegenerative diseases. This section briefly explores their relationship and interactions.

### Alzheimer’s disease

AD is a neurodegenerative disease associated with the deposition of amyloid plaques and neurofibrillary tangles, formed by Aβ peptides and phospho-Tau, respectively, in the CNS [140]. AD is a devastating neurological disorder that progressively impairs cognition and memory and severely affects the quality of life and well-being of patients, family members, and caregivers [141]. Approximately 2% of AD cases are caused by familial AD, whereas the remaining 98% are caused by sporadic AD. Considerable evidence indicates that AD pathogenesis is multifactorial. Some mechanisms related to AD are known, such as intracellular and extracellular Aβ fibrillary aggregates, Tau phosphorylation, over-activation of NMDARs, oxidative stress, and neuroinflammation [142–144].

Previously, it was believed that the pathology of AD was mainly due to protein misfolding and aggregation. Recent studies have challenged the traditional pathogenic mechanisms. In AD model mice, defects in autophagic lysosomal (AL) acidification precede Aβ deposition, and the accumulation of Aβ and amyloid beta precursor protein-C-terminal fragment in insufficiently acidified AL forms a PANTHOS [(poisonous anthos (flower))] pattern and develops into senile plaques, underscoring the critical role of AL dysfunction within neurons [145]; Aβ and Tau act synergistically throughout the course of AD, and combined therapies targeting both may be the key [146]; there are also views that AD is an autoimmune disease, and that Aβ is regarded as a cytokine and immune peptide with both immunomodulatory and antimicrobial properties. Its antimicrobial properties can lead to a chronic autoimmune cycle caused by erroneous attacks on “self” neurons, and its immunomodulatory properties affect microglia function, which ultimately leads to neuronal death [147]. The apolipoprotein E4 genotype contributes to the abnormal accumulation of lipid droplets (LD) in microglia and the formation of neurotoxic LD-accumulating microglia, which are capable of directly inducing neuronal Tau phosphorylation and apoptosis, and the degree of LD accumulation is positively correlated with the severity of cognitive decline and pathology [148]. Dysbiosis of the intestinal flora also affects the process of AD [149], and transplantation of a healthy microbiota improves the cognitive deficits and reduces Aβ and Tau deposition in AD model mice [150]. In conclusion, the pathological mechanisms of AD are complex, and the new understanding brings new directions for its research and prevention.

Excitotoxicity also plays an integral part in the pathogenesis of AD. A lot of evidence has shown that excessive activation of NMDARs in AD is related to changes in the expression of calpain, Ca^2+^/CaMKII, GSK-3β, and other proteins, which play crucial roles in excitotoxicity and neurodegeneration [151]. Researchers have recently observed a significant protective effect against KA-induced excitotoxicity and hippocampal superoxide production in vivo in Tau gene knockout mice [144]. NMNAT2 is beneficial to improve cognitive function, and it colocalizes with the aggregated Tau in the brains of patients with AD so that the misfolded protein can be effectively identified [152]. There is research that the human syndrome is a neurodegenerative disorder resulting from Tau-mediated excitotoxic neurodegeneration. Furthermore, the increased Tau protein and excitotoxicity in methyl-CpG-binding protein 2 neurons and astrocytes may be caused by an imbalance in glutamate homeostasis [153]. Furthermore, sigma 1 receptor (σ1R) is involved in excitotoxicity. The σ1R ligand mediates the generation of new synapses and dendritic spines through the mitogen-activated protein kinase/extracellular regulated kinase and PI3K/AKT signaling pathways, thereby exerting neuroprotective effects [154]. In conclusion, the pathological mechanisms of AD are complex, and the new understanding brings new directions for its research and prevention.

### Parkinson’s disease

PD is a common systemic neurodegenerative disease. When 50%–60% of dopamine (DA) neurons in the substantia nigra are lost [155], unilateral static tremor, dyskinesia and rigidity will highly likely develop. Clinical non-motor symptoms mainly include cognitive impairment, constipation, and sleep disorders. The pathological features of PD are the degeneration of nigral DA neurons caused by the appearance of Lewy bodies and the aggregation of α-synuclein (α-syn). The accumulation of α-syn in DA neurons into higher molecular pathological structures causes neurodegeneration, which further affects autophagy/lysosomal balance, mitochondrial homeostasis, ER, synaptic function, oxidative stress, and excitotoxicity [156]. α-syn can be degraded via the ubiquitin-proteasome system and autophagy/lysosomal pathways, but protein clearance is impaired in PD, resulting in abnormal protein accumulation. In addition, α-syn pathological changes can spread in the brain, with multiple routes of transmission with unknown initiating factors [157, 158]. Multiple factors contribute to mitochondrial complex I dysfunction, affecting energy metabolism and exacerbating oxidative stress, which damages neurons, and PINK1/Parkin-mediated mitochondrial autophagy is disturbed by mutations in PD-associated genes and aberrant expression of deubiquitinating enzymes, imbalances in mitochondrial dynamics, and aberrant interactions with other organelles, which all contribute to the progression of PD [159].

The pathological mechanisms of the immune response in PD involve multiple alterations and interactions between the central and peripheral immune systems. Microglia and astrocytes in the CNS are activated to release inflammatory factors and damage neurons, and in the peripheral immune system, alterations in blood immune cell subsets and function, and elevated inflammatory factors correlate with central inflammatory and neurodegenerative changes, but the consistency is influenced by clinical phenotype and disease stage [160, 161]. Gastrointestinal dysfunction is prominent in PD, with symptoms such as constipation preceding motor symptoms and influencing disease progression. α-syn or transmission from the gut to the brain via the vagus nerve affects neurons, and the gut microbiota is altered in patients with PD and is associated with disease and influenced by diet [162, 163].

The pathogenesis of excitotoxicity in PD is complex, and its treatment strategies are multifaceted. These aspects of PD have thus attracted significant research interest. Excessive glutamate release activates NMDARs and AMPARs, leading to Ca^2+^ overload, promoting neuronal oxidative stress, inducing mitochondrial damage, and increasing the risk of cell death. In addition, because DA neurons in the substantia nigra pars compacta are particularly susceptible to oxidative stress, excessive activation of glutamate receptors exacerbates ROS production and free radical levels, which is vital to the pathogenesis of PD. As a glutamate uptake inhibitor, L-*trans*-pyrrolidine-2,4-dicarboxylate can produce sustained low-level excitotoxic damage [164, 165]. Using whole-cell patch-clamp electrophysiology and micro-fluorescence Ca^2+^ measurement of rat brain slices, study has demonstrated that the inhibition of hyperpolarization-activated current (Ih) determines the synaptic excitability of DA neurons. Furthermore, Ih inhibition enhances the AMPA/NMDAR-mediated response, indirectly regulates voltage-dependent Ca^2+^ influx, and weakens GABA_A_ receptor activity [166]. Increasing evidence shows that some DA neurons express vesicular GLT 2 (VGLUT2). VGLUT2 can maintain mitochondrial homeostasis and reduce intracellular ROS production. The glutamate co-release of DA neurons expressing VGLUT2 drives the burst discharge of cholinergic interneurons, stimulates the nicotinic acetylcholine receptor at the end of DA neurons, and promotes DA/glutamate co-release, forming a positive feedback loop [167]. A recent study has demonstrated the dynamic relationship between Parkin and mitochondria and the ER under excitotoxicity. They exposed neurons to acute glutamate, causing excitotoxicity, which promoted Parkin to move to the mitochondria, ER, and mitochondrial-ER junctions [81]. The interconnectedness of these factors drives the progression of the disease, and although some results have been achieved, in-depth investigations are still needed to clarify the mechanisms and guide treatment.

### Huntington’s disease

HD is caused by the abnormal elongation of CAG trinucleotide repeats in the Huntington (*HTT*) gene, resulting in mutations in the HTT protein, which causes the gradual development of this fatal hereditary neurodegenerative disease. mHTT disrupts multiple signaling pathways and interactions within neurons, causing the immature development of medium spiny neurons in the striatum and synaptic dysfunction.

Chronic 3-NP administration depletes ATP and NAD^+^ levels and increases the release of ILs, TNF-α, glutamate, and iNOS [168, 169]. Excitotoxicity activates cysteine aspartic proteases, which participate in NMDA-mediated apoptosis. NMDA was used to induce acute injury in primary rat cortical neurons, which markedly increased *HTT* mRNA expression within a short time, as well as increased the expression of *caspase-6*, *caspase-3*, and *caspase-8* [92]. In primary neurons under excessive stimulation by NMDARs, kinase D-interacting substrate of 220 kDa exon 33 (Kidins220-C33) was downregulated by protease calpain activation and was mainly expressed in neurons. Furthermore, Kidins220-C33 was selectively downregulated in the brain tissue of HD mice [170]. DAPK1 is a potential target for synaptic protection in HD, which can prevent the loss of striatum spines. Inhibition of DAPK1 expression promotes excitotoxicity [171]. In the 3-NP-induced HD mouse disease model, the nuclei surrounded by obvious focal diffuse gliosis were observed around the striatum and hippocampal brain slices in the 3-NP group. In contrast, the cell membrane in the forskolin (FSK) group remained intact, and the cellular nucleus showed no visible changes. More importantly, FSK mitigated excitotoxicity and improved the learning ability and memory in mice [76].

### Amyotrophic lateral sclerosis

The molecular mechanism of excitotoxicity in ALS involves the excessive release of glutamate, causing Ca^2+^ overload and excessive activation of the glutamate receptor AMPA, thereby inducing astrocyte proliferation, mitochondrial dysfunction, and energy exhaustion. The chronic excitotoxicity generated by these cascade reactions gradually causes the death of spinal cord MNs (SMNs) and eventually leads to paralysis. Although the above mechanisms have been discovered and confirmed repeatedly, therapeutic strategies should be more specifically targeted to mitochondrial energy defects rather than the neuroprotective effects of antioxidants [52]. Some reports have indicated that the decrease in excitatory amino acid transporter 2 (EAAT2) in the ALS mouse model and human SMNs significantly reduces the expression of membrane proteins [172].

Glutamate-mediated excitotoxicity induces Ca^2+^ overload; Ca^2+^ promotes the translocation of ALS-related RNA-binding proteins from the nucleus to the cytoplasm in primary cortical neurons and MNs. In turn, the translocation and translation of FUS promote the expression of AMPA-type subunit 2 (*Gria2*) mRNA in dendrites [173]. DL-Threo-*β*-benzyloxyaspartate (TBOA) is a glutamate uptake blocker frequently used to induce excitotoxicity. Activation of nicotinic acetylcholine receptors can suppress the downstream mechanisms of excitotoxicity, including intracellular Ca^2+^ overload, increased ROS, and mitochondrial energy metabolism disorders. Nicotine significantly reduced TBOA-induced bursting activity, inhibited synaptic excitability transmission, and increased hypoglossal motoneuron survival [174]. Overactive somatostatin (Sst) interneurons caused the continuous de-inhibition of layer 5 pyramidal neurons (L5-PN) in TDP-43^A315T^ mice and significant reductions in miniature and evoked inhibitory postsynaptic currents. On the contrary, the focal ablation of Sst interneurons reduced the excitotoxicity and GABAergic synaptic strength of L5-PN [175]. Lysophosphatidic acid inhibits SMN survival and increases MN intrinsic membrane excitability through lpa_1_ and TWIK-related acid-sensitive K^+^ subunit 1. However, administration of siRNA_*lpa1*_ or AM095 (a lpa_1_ inhibitor) mitigated MN injury and protected neurons in SOD1^G93A^ mice, which thus identifies a potential target in the treatment of ALS [172].

## Combatting excitotoxicity in neurodegeneration

We briefly illustrated the relationship between excitotoxicity and neurodegenerative diseases in the previous section. It is vital to prevent excitotoxicity from causing damage in patients with neurodegenerative diseases. Increased glutamate release, dysfunction of GLTs, and abnormal expression of glutamate receptors can lead to excitotoxicity. In view of the mechanism of excitotoxicity, a lot of inhibitors related to excitotoxicity appear in our field of vision.

### Glutamate receptor antagonist

In recent years, research into the inhibition of excitotoxicity has predominantly centered on the development of glutamate receptor antagonists. Chronic hypoxia may lead to cognitive dysfunction by increasing the expression of NMDARs and increasing the level of oxidative stress. After administration of UB-ALT-EV in 5xFAD mice, it was found that the deposition of Aβ and the phosphorylation of Tau protein in the brain region of mice were significantly reduced. Interestingly, UB-ALT-EV reduced the expression of Ca^2+^-dependent protein calpain 1 compared with memantine [176–178]. Acamprosate is a very common therapeutic drug in patients with chronic alcohol dependence. Studies have demonstrated that acamprosate can produce a sustained neuroprotective effect in stroke patients. This is because acamprosate can indirectly inhibit NMDAR activity, inhibit calpain activity, and increase signal transducer and activator of transcription 6 abundance. Moreover, acamprosate is well tolerated and has potential value in adjuvant stroke treatment [179].

RNA aptamers are potential therapeutic agents as antagonists of iGluRs. By constructing chemically modified RNA libraries, full-length aptamer FN1040 and its truncated aptamer FN1040s were screened by systematic evolution of ligands by exponential enrichment technology, which inhibited different glutamate receptor subtypes. FN1040s selectively inhibited GluA1 and GluA2, and FN1040 additionally inhibited GluK1 and GluN1a/2A and GluN1a/2B, both of which had similar potency and good stability, providing a potential tool for the study of glutamate receptor function and the treatment of related neurological diseases [180]. FB9s-b and FB9s-r are chemically modified aptamers derived from the study of the dual-activity RNA aptamer AB9s. FB9s-b selectively inhibits GluK1, GluK2 and its heterodimer with GluK5, and FB9s-r inhibits AMPAR, and the stability of both of them was significantly improved by the 2′-fluorine modification, and they retained the selectivity for their respective receptors and similar inhibition potency as before, and maintained the selectivity for the respective receptors and the inhibitory potency similar to that before the modification [181].

Epilepsy induces the upregulation of mGluR5 expression, and reducing the expression of mGluR5 may be a potential strategy for the treatment of epilepsy. 3-[(2-methyl-1,3-thiazol-4-yl) ethynyl]-pyridine (MTEP), a selective mGluR5 antagonist, administered during the latency of the mouse model, significantly rescued neuronal loss and hippocampal astrocyte proliferation in mice. However, administration of MTEP could not prevent the development of epilepsy. This indicates that the regulation of mGluR5 alone is ineffective for the treatment of epilepsy [182]. The mGluR5 antagonist 2-methyl-6-(phenylethynyl) pyridine reduced the expression of CREB, p-ERK, and Rad51 in the rotenone-induced PD rat model and alleviated the DNA damage of dopaminergic neurons [183].

UBP-310 is a KAR antagonist with high selectivity for the GluK1 subunit, which reduces desensitization of the GluK1/GluK2 heterodimer and eliminates the desensitization of GluK1/GluK5 heterodimer [184]. In an acute mPTP mouse model of PD, administration of UBP-310 significantly increased the number of dopaminergic and total neuronal survival in the substantia nigra compacta [185]. Compound 28 is a new quinoxaline-2,3-dione derivative synthesized from N-[2,3-dioxo-6-(phenylethynyl)-3,4-dihydroquinoxalin-1(2H)-yl] benzamide as a lead structure, which is a competitive GluK3 antagonist with sub-micromolar affinity and unprecedentedly high binding selectivity, with 400-fold higher selectivity for GluK3 than for other cognate receptors, and no agonistic or antagonistic activity in mGluRs isoforms [186].

### Natural compounds

The therapeutic effects of natural compounds are diverse. γ-Oryzanol is a natural compound rich in phytosterols, which has anti-inflammatory and anti-cancer effects. In glutamate-exposed HT22 cells, γ-Oryzanol improved Δ*Ψ*_m_, alleviated GSH depletion, and reduced the expression level of CAMKII, thereby protecting neurons from glutamate excitotoxicity [187]. Bidentatide is a 33-amino-acid peptide extracted from *Achyranthes bidentata* Blume, which has the effect of inhibiting NR2B. Mainly by inhibiting Ca^2+^ in neurons, NMDA current intensity, and apoptosis-related proteins [188]. In the study of curcumin-based non-competitive AMPAR antagonists, it was found that nitrogen affects the binding of receptors in the spillover loop, and both of which can reduce the activation current of AMPA [189]. Studies have found that the combination of L-theanine, *Magnolia officinalis*, and *Melissa officinalis* showed neuroprotective effects against excitotoxicity in vitro and anti-anxiety and antidepressant activities in vivo [190]. DT-010 is a derivative of danshensu, and DT-010 almost completely inhibits the accumulation of intracellular ROS caused by glutamate stimulation of primary hippocampal neurons. DT-010 enhances neuronal activity by activating the PI3K/AKT/GSK-3β pathway [191].

### Other inhibitors

In addition to the common glutamate receptor antagonists and natural compounds, there are some excitotoxic inhibitors. In the 6-OHDA-mediated PD model, the oxidative stress sensor peroxisome proliferator-activated receptor β/δ (PPARβ/δ) may be activated, resulting in a sharp decline in cell viability. PPARβ/δ antagonist GSK0660 rescued the decrease of brain-derived neurotrophic factor and tropomyosin receptor kinase B expression and reduced oxidative stress by activating CREB [192]. Capsaicin is an agonist of the transient receptor potential vanilloid 1 receptor. Pretreatment of capsaicin reduces the levels of pro-inflammatory cytokines IL-1β and IL-6 in brain tissue stimulated by ibotenate, and inhibits the activity of brain mast cells in a dose-dependent manner [193]. After soman-induced seizures, the expression of transient receptor potential vanilloid 4 (TRPV4) in the hippocampus of rats was significantly up-regulated. The TRPV4 antagonist GSK2193874, as a preventive treatment, inhibits NMDAR-mediated excitotoxicity and NLRP3 inflammasome activation, and reduces the seizure rate [194]. Vafidemstat (ORY-2001) is a clinical-stage lysine-specific histone demethylase 1A inhibitor that is being developed for the treatment of neurodegenerative and psychiatric diseases. ORY-2001 reduced neuronal glutamate excitotoxic damage and improved learning and memory deficits in rodents. More importantly, compared with FTY270 approved by the FDA for the treatment of chronic MS, ORY-2001 does not target σ1R, does not have bradycardia, and does not cause gastrointestinal toxicity [195]. Our study found that NADPH could decrease KA-induced p62 and LC3-II upregulation. Moreover, NADPH effectively mitigated the neuronal loss caused by KA [122].

Iststradefylline is a clinically approved adenosine A2A receptor antagonist. Iststradefylline effectively reduces the excitotoxic damage of NMDA/KA-treated rat cochlea and rescues spiral ganglion neurons’ neurites [196]. Protease-activated receptor 4 (PAR4) is a low-affinity thrombin receptor, which is up-regulated in stroke. PAR4 antagonist ML354 inhibited OGD-induced increase in Ca^2+^ activity and decreased Bax/Bcl-2 ratio, reducing neuronal excitotoxicity [197–199]. The glucocorticoid receptor agonist dexamethasone reduced the activity of retinal microglia and inhibited the loss of retinal neurons caused by excitotoxicity [200]. Non-steroidal anti-inflammatory drugs COX inhibitors, especially COX-2 inhibitors, can protect neurons from excitotoxicity damage [201]. Dehydroepiandrosterone is a σ1R agonist, which significantly increases the activity of GLT-1 in astrocytes and its transport to the cell surface [202, 203]. In general, it is very important to inhibit or slow down the progression of excitotoxicity in neurodegenerative diseases. However, due to the complexity of the disease and the singleness of drug targets, it is difficult to develop excitotoxicity antagonists in clinical practice.

## Conclusions and future perspectives

The effects of glutamate-mediated excitotoxicity on disease are extensive and profound. Membrane potential disruption destabilizes Na^+^/K^+^ gradients across neuronal and glial membranes, triggering EAATs to switch from glutamate uptake to reverse transport. Concurrent ionic concentration shifts generate the driving force for glutamate efflux, resulting in excessive synaptic glutamate accumulation [22]. Excessive excitatory neurotransmitter glutamate is released in the synaptic cleft, glutamate receptors are over-activated, and Ca^2+^ flows into neurons, triggering ion homeostasis imbalance, oxidative stress, and mitochondrial dysfunction. Under normal physiological conditions, neuronal action potentials are transmitted to presynaptic terminals, causing voltage-gated Ca^2+^ channels to open and extracellular Ca^2+^ to flow in, prompting synaptic vesicles to release glutamate into the synaptic cleft, where glutamate binds to glutamate receptors on the postsynaptic membrane, triggering the influx of cations into the postsynaptic neuron and altering the membrane potential to achieve signal transmission. At the same time, GLTs use electrochemical gradients to transport glutamate into the cell and maintain its stable concentration. However, in pathological or abnormal physiological states, the equilibrium is disturbed. For example, during excitotoxicity, the intracellular Ca^2+^ concentration is abnormally elevated, leading to an excessive release of glutamate and a massive influx of Ca^2+^ into the postsynaptic neuron, triggering an imbalance in Ca^2+^ homeostasis and resulting in intracellular Ca^2+^ overload. This leads to mitochondrial metabolic disorders, affecting ATP production, activating kinases to produce O_2_^-^, triggering oxidative stress, and further damaging mitochondria [204]. It also disrupts ER Ca^2+^ pool homeostasis, triggering ERS, interfering with protein degradation, and leading to protein accumulation. Altered levels of NAD^+^ exacerbate oxidative stress and mitochondrial metabolic disorders.

Multiple forms of cell death exist in neurodegenerative diseases, such as autophagy, apoptosis, ferroptosis, and necrosis. These mechanisms, although they play different roles in different situations, are essential for maintaining metabolic homeostasis and neuroprotection in the body. It is urgent to alleviate the serious damage caused by excitotoxicity in neurodegenerative diseases. Moreover, it is important to continue investigating the underlying molecular mechanism between excitotoxicity and neurodegenerative diseases. Nerinetide is an inhibitor of the protein–protein interaction of PSD-95. Studies have shown that in the MCAO rat model, nerinetide administration before alteplase can maintain its effectiveness [205]. In patients without alteplase, nerinetide was associated with improved outcomes. After administration of empagliflozin in patients with type 2 diabetes, the concentration of glutamate and its precursor glutamine decreased. Study has found that ceftriaxone can delay the onset of ALS by activating EAAT2 [206].

In conclusion, the emphasis should be placed on the research and development of efficacious, multimodal pharmaceuticals that target the intricate complexities of the nervous system. This treatise provides an exhaustive review of the scholarly discourse surrounding excitotoxicity over the past decade, aiming to elucidate the nexus between excitotoxicity and the pathogenesis of neurodegenerative disorders, thereby potentially catalyzing novel avenues for the invention of neuroprotective therapeutics to counteract excitotoxicity.
